# Supplementary material for: Are carbon emissions trading and green financial instruments synergistic? -Comprehensive quantitative research based on content analysis
Source: PLoS One. 2024 Mar 7;19(3):e0298601. doi: 10.1371/journal.pone.0298601 (PMC10919601; doi:10.1371/journal.pone.0298601)
Supplement: S1 Data — (ZIP) [file pone.0298601.s001.zip › Data/地级市Z_F工作报告.zip!/║ú─╧/║ú┐┌╩╨/└┤╫╘╣1⁄2╓┌║┼í╛┬φ┐╦╩2╛▌═°í┐_2019.docx]

各位代表：

　　现在，我代表海口市人民政府向大会报告工作，请予审议，并请各位政协委员和列席人员提出意见。

　　2018年工作回顾

　　2018年是我市发展极不平凡的一年。4月13日，习近平总书记亲临海口视察指导，出席庆祝海南建省办经济特区30周年大会并发表重要讲话（以下简称“4·13”重要讲话），中央出台支持海南全面深化改革开放的指导意见（以下简称中央12号文件），支持海南全岛建设自由贸易试验区，逐步探索、稳步推进中国特色自由贸易港建设，赋予海南全面深化改革开放新的重大责任和历史使命，为海口高质量发展指明了方向，给全市上下以极大的鼓舞。

　　一年来，在省委省政府和市委的坚强领导下，在市人大、市政协的监督支持下，我们深入学习贯彻习近平总书记“4·13”重要讲话和中央12号文件精神，以习近平新时代中国特色社会主义思想为指导，增强“四个意识”、坚定“四个自信”、践行“两个维护”，坚持稳中求进工作总基调，担当实干、攻坚克难，经济社会发展与生态治理有机结合、实现“双赢”，主要经济指标高于全省，较好地完成了市十六届人大四次会议确定的各项任务。全年实现地区生产总值1511亿元，同比增长7.6%；固定资产投资完成1313亿元，同比下降6.2%；地方一般公共预算收入169.9亿元，同口径增长13.9%；社会消费品零售总额757.6亿元，同比增长5.9%；城镇常住居民人均可支配收入36137元，同比增长8.5%；农村常住居民人均可支配收入14886元，同比增长8.2%。空气质量在全国169个城市中排名第一，全面完成省下达的节能减排降碳控制目标。

　　（一）自贸区建设取得良好开局

　　江东新区高起点规划建设。聚焦“三区一中心”集中展示区目标定位，坚持规划先行，全球超过60家一流设计团队踊跃参与江东新区概念规划征集，总体规划纲要、起步区城市设计、临空产业园规划等高质量完成，路网、水系管网、建筑风貌、智慧城市等配套规划协同推进。着力夯实基础工作，积极有效争取国家部委支持，以超常规力度完成土地、人口、海洋、地质等17项基础调查；严控项目建设，严管户口迁入，实现新增违建为“零”，拆除、处置存量违建299.6万平方米；把党支部建在一线，60天完成9600亩土地征收。着力完善基础设施，启动绕城高速（二期）、文明东越江隧道、白驹大道延长线等9个首批基础性、先导性、示范性重大工程，海口首家国际学校哈罗公学开工建设，江东大道实现功能性通车。着力培育基础产业，深化产业发展定位和规划研究，严格项目准入门槛，49家大型知名企业申请落户。

　　重要片区开发加紧推进。“一园两城两区”产业集聚主阵地初具雏形。临空产业园加快推进，构建“政府主导、企业主力”新机制，顺丰、菜鸟、圆通等快递巨头相继进驻，航空维修、航食加工等项目率先落地。新海生态临港新城规划优化提升，客运综合枢纽全面动工。美安科技新城与上海临港开展全方位合作，一批高新技术产业项目加快建设。

西海岸南片区完成控规修编，复兴城互联网总部基地启动，未来产业园吸引36家知名移动互联网企业意向入园。海口湾片区首推建筑师负责制，对不符合规划风貌管控的建设项目一律暂停、优化，强力处置闲置建设用地。

　　重点项目成效明显。集中开展“重点项目服务季”，强化“六个一”责任制，设立市区两级指挥部，责任到区、党政同责，“一天当作三天用”推动项目建设。全年实施省市重点项目55个，完成投资270亿元，超年度计划9个百分点；四季度固定资产投资连续3个月环比增长40%以上。市民游客中心等一批民生项目建成使用；“海澄文”联通工程等一批基础设施项目加快推进。成功承办海南自由贸易试验区建设项目“11·28”“12·28”集中开工活动，海南国际会展中心（二期）、海口城际公交化铁路、海秀快速路（二期）等38个项目开工建设，总投资418亿元、占全省30%，扛起了项目建设的省会担当。

　　招商引才成果丰硕。深入开展百日大招商，赴北京、上海、香港等地点对点精准招商，创新开展第三方委托招商，外资招商、央企招商、优强企业招商取得历史性突破。引进世界500强企业4家、行业领军企业20家、知名品牌企业23家，世界四大会计师事务所全部进驻；新增外资企业93家、增长1倍，实际利用外资2.5亿美元、增长7.8倍；首批认定总部经济企业22家，占全省73%，中旅集团成为首家落户海南的央企一级总部。全面落实“百万人才进海南行动计划”，实施5项人才引进计划、7项人才培养工程，全年引进各类人才1.49万人，占全省50%左右。

　　营商环境不断优化。持续深化“放管服”改革，“极简审批”模式获国务院通报表扬并向全国推广。网上办事大厅加快建设，“12345+营商服务”模式向全省推广。国际贸易“单一窗口”上线运行，整体通关时间压缩近半。海口爱奇艺知识产权供应链资产支持证券成功发行，成为全国首例知识产权资产证券化项目。工商注册全城通办，“二十证合一”升级为“三十一证合一”。严格落实结构性减税政策，设立市级中小企业发展专项资金，成立服务民营经济工作专班。营商环境基础设施在全国36个大中城市中位列第二，入选中国最具投资潜力城市50强，全年新增市场主体6.18万户、注册资金3848亿元，分别增长20%、63.4%。

　　对外开放不断扩大。实施59国入境旅游免签，新开通海口至悉尼、莫斯科等国际航线15条，美兰机场境外旅客吞吐量113万人次、增长29%，创历史新高。海口至越南、菲律宾邮轮旅游航线开通，邮轮运营航次全省第一。获批国家跨境电子商务综合试验区和平行进口汽车试点，创新推出自贸区文化保税、融资租赁等“十项任务清单”。全年外贸进出口总额341.2亿元，同比增长62.3%，占全省40.2%。深化与“一带一路”国家交流合作，举办第十一届海口—东盟国家驻广州领事馆对话会，新增菲律宾公主港等3个国际友好交流城市。琼州海峡经济带建设加快推进，成功举办北部湾经济合作组织暨城市合作组织会议，与湛江全方位、多层次交流互动，交通港航、环境保护、菜篮子等领域合作不断深化。

　　（二）产业发展迈出重要步伐

　　“旅游+”产业融合发展。桂林洋国家热带农业公园、观澜湖狂野水世界、长影环球100等项目开业运营，水上飞机、游艇观光航线开通，15个夜市街区完成升级改造，成为海口旅游观光新名片。五源河文体中心体育场建成，中国足球（南方）训练基地迎来国家队首训，全球首家巴萨世界主题馆、全国首家美国职业篮球联赛（NBA）互动体验馆相继开馆，亚洲最大的国家帆船基地公共码头建成开港，中国帆船帆板运动协会南方总部基地挂牌；中国—巴勒斯坦足球友谊赛、中国帆船联赛总决赛、“万人竹竿舞”“火山音乐节”等文旅结合的赛事活动成功举办，旅游城市知名度加快提升。举办上规模会议和展览活动315场、增长17%，综合收入122亿元、增长18.4%，获评中国最具品牌会奖价值目的地和最具影响力会展目的地。离岛免税购物政策再升级，实现免税品销售22亿元。全年共接待过夜游客2258万人次，旅游总收入298亿元，分别增长11%、12%，实现旅游人数、效益“双提升”。

　　现代产业体系加快培育。金融业稳步发展，浦发银行海南离岸金融创新中心挂牌成立，国投融资等4家融资租赁公司开业运营，国铁融资完成首单动车组租赁业务。互联网产业实现相关营业收入235亿元，增长14.5%；全省首个5G基站开通，蚂蚁金服、字节跳动等行业巨头成功引进，复兴城互联网创新创业园税收同比增长20倍。入选国家物流枢纽布局承载城市，空中全货机航线开通，海口港完成货物吞吐量10765万吨、集装箱吞吐量185万标箱，分别增长6.4%、12.9%。电子商务实现交易额1356亿元，增长22.3%，占全省70%；线下移动支付覆盖率从年初的40%提升至90%以上，位居全国前列，盒马鲜生等新零售业态加快布局。医药制造稳步增长，新增销售过亿元单品40个，实现产值229亿元、增长16％。海洋经济创新发展，新增公共服务平台7个。打造创业创新聚集地30家，高新区获评国家小微企业创业创新示范基地。入围国家知识产权运营服务体系建设重点城市，全年新增高新技术企业72家，总数达270家、占全省75%。

　　（三）生态宜居品质持续提升

　　生态治理成效显著。坚决落实中央环保督察和国家海洋督察问题整改，全年查处环境违法案件433宗，污染防治攻坚战取得阶段性胜利。出台《生活垃圾分类管理办法》《湿地保护若干规定》，划定畜禽禁养和限养区域并关停养殖场349家；强力查处非法采砂运输车辆及装载设备1722辆，抓获犯罪嫌疑人95人，非法采砂行为得到有效遏制。全年拆除、处置违法建筑692万平方米，超额完成省下达任务。实施小客车保有量调控和配额指标管理，淘汰黄标车2677辆，推广新能源汽车12127辆。完成城市集中式饮用水源地环境整治，饮用水源地国控断面达标率、近岸海域水质达标率均达100%；河长制湖长制系统测评位列全省第一，建成区21个水体全部消除黑臭，美舍河、五源河荣登全国城市水体治理光荣榜，海口水环境治理在国务院第五次大督查中被列为典型经验通报表扬。特别是全市上下再接再厉，成功获评全球首批国际湿地城市，成为海口建设国际化滨江滨海花园城市的又一里程碑。

　　乡村振兴统筹推进。农业生产结构不断优化，新增荔枝、莲雾等高效作物8000亩，花卉种植突破7.2万亩，全年实现农业增加值64亿元，增长4.5%。建设特色产业小镇10个、全省星级美丽乡村41个，光纤和4G网络覆盖全部行政村，完成农村道路1286公里、危桥改造28座、安全饮水工程385宗、危房改造1180户、改厕7400户，首批117个自然村生活污水治理工程加快实施，全省推进百镇千村建设现场会在秀英施茶村召开。完成农村土地承包经营权确权登记颁证，农村集体产权制度改革琼山试点任务全面完成并在全市铺开。总规模1.8万亩的龙湾复垦项目全面启动。举办“火山荔枝月”活动，打响火山荔枝品牌，销量和收入分别增长39.5%、64.8%，实现丰产丰收。“冬交会”签订农产品订单45.2亿元、增长20.8%，创历年新高。

　　城市更新扎实推进。成立由院士、建筑大师组成的城市设计和建筑风貌专家委员会。城市更新首批示范项目全部竣工，仅用120天完成三角池片区综合整治，延续了“闯海人”的城市记忆。琼台福地和鼓楼保护修缮、万绿园景观功能提升工程加快推进；“琼州第一塔”明昌塔启动复建，红城湖公园全面动工。完成棚户区改造5863户，超额30%完成省下达任务。实施重要门户和主干道路“五化”提升，依法拆除广告牌匾6278块，立体绿化人行天桥11座，完成311栋楼体、33.8公里市政道路绿化带亮化，全国文明城市和国家卫生城市成果巩固提升，美丽海口8次入镜央视新闻联播，尽显椰城魅力。

　　（四）民生福祉得到明显增进

　　全年民生支出达171.9亿元，占全市一般公共预算支出的72.4%。

　　精准脱贫深入推进。建立从市到自然村的四级战斗体系，完成农村人口拉网式大排查，实现贫困家庭应纳尽纳“零漏评”、应退尽退“零错评”；投入4539万元实施精准帮扶，实现产业组织化全覆盖、零就业贫困家庭全清零、“三保障”政策全落实，圆满完成年度减贫任务，获得全省打赢脱贫攻坚战大比武第一名。

　　民生保障全力抓实。抓好就业保障，转移农村劳动力就业7430人，新增城镇就业3.36万人，城镇登记失业率1.34%。退休人员基本养老金、城乡居民基础养老金以及居民基本医保财政补助标准稳步提高。强化民生兜底，23923名低保、优抚和特困人员基本生活得到保障。新增公共租赁住房717套、实施租赁货币保障3600户；集中解决房产“办证难”历史问题67宗，办证23万平方米。严格执行房地产调控系列措施，有效遏制房价过快上涨。全面落实地方粮食储备，用心用情做好“菜篮子”保供稳价，密集开展市场巡查执法2100余人次，试点推行蔬菜直销模式，新增“菜篮子”公益性摊位56个，覆盖全市所有农贸市场，有效平抑市场菜价。

　　公共服务明显改善。实施公办中小学校（幼儿园）三年建设计划，侨中美丽沙分校、人大附中、北大附小及8所幼儿园建成使用，新增学位9970个，长彤学校等4所学校开工，创建湿地学校6所。新建中小学游泳池21个，3万余名中小学生参加游泳培训。美兰区、秀英区通过国家慢性病综合防控和健康促进示范区评审；市属公立医院全面取消药品加成，继续实施“先看病后付费”，累计受益人数达9万人；全面完成15万学生眼疾病和1.1万妇女“两癌”免费筛查；启动272家基层医疗机构标准化建设。新增公交线路26条，荣获全国智慧交通用户服务创新奖。创新居家养老服务模式，在6个社区启动“长者饭堂”助餐服务试点。举办全民健身和文化惠民活动503场，海口创作出品电影《旋风女队》荣获中国电影华表奖，《黎族家园》入选国家舞台艺术精品剧目，《海口年鉴（2017）》荣获全国地市级综合年鉴一等奖。勇夺全省第五届运动会和第七届残运会总分第一。

　　社会治理创新发展。“城市大脑”框架基本成型，大数据在社会治理、防灾救灾中的作用进一步加强。“12345+网格化”智慧平台构建城市综合治理新格局，荣获全国“先锋奖”“金数奖”和“金铃奖”；“椰城警民通”上线，在全国率先实现警务服务“移动办”。龙华区获评全国法治创建先进单位，秀英区东方洋获评全国优秀社区，琼山区、美兰区入选全国新时代文明实践中心建设试点。“美舍河变形计”荣获全国青年志愿服务项目大赛金奖。毒品治理跻身全国36个大中城市前三，全国禁毒重点整治示范创建暨宣传教育工作会议在海口召开；深入开展扫黑除恶，打掉涉黑涉恶犯罪团伙28个，包括“砂霸”团伙3个、“运霸”团伙1个，得到省委省政府肯定。安全生产形势持续向好，事故起数和死亡人数分别下降6.8%、23.8%，全年没有发生导致人员伤亡的火灾事故。扎实推进国家食品安全示范城市创建，全市75%的餐饮店完成“明厨亮灶”。绿色殡葬改革成效明显。积极开展海口舰“回家”等双拥共建活动，成功举办首次“海澄文”人民防空协同演练。在春节“抗雾保运”中，全城动员，一级应急响应148小时，全程全方位提供保障服务，温暖了73.4万旅客的回家路，得到社会各界高度评价，充分展示了海口市民的文明素质、干部的良好作风和城市的文明程度。

　　（五）政府效能稳步提升

　　全市政府系统深入开展“勇当先锋、做好表率”专题活动，狠抓执行力建设，强化对重要任务事项全程跟踪问效，行政效能明显提升。严格落实全面从严治党要求，扎实抓好省委巡视反馈问题整改，稳步推进国资国企改革，强化对关键领域和关键环节审计，严肃查处了一批违纪违法案件，“四风”问题有效纠治，“三公”经费下降40.3%。主动接受市人大法律监督和工作监督、市政协民主监督及社会舆论监督，提请市人大常委会审议法规议案2件，制定、修改、废止政府规章2件，办理答复人大代表建议208件、政协提案410件，办结率100%。

　　此外，统计、气象、三防、地震、保密、档案、科协、社科联、工商联、民族宗教、外事侨务、海防口岸、对台事务等工作取得新进展，工会、青少年、妇女儿童、老龄、残疾人、慈善等事业取得新进步。

　　各位代表！过去一年，外部环境复杂严峻、经济下行压力持续加大、经历产业转型培育阵痛，取得这些成绩殊为不易！我们深刻体会到：这是党中央国务院、省委省政府和市委坚强领导的结果，是市人大、市政协及各位代表、各位委员支持的结果，是全市227万人民群众攻坚克难、共同奋斗的结果。在这里，我谨代表市人民政府，向全市人民，向人大代表和政协委员，向各民主党派、工商联和社会各界人士，向驻市部队、武警官兵和公安司法干警，向关心支持海口发展的港澳台同胞、广大海外侨胞及国际友人，表示崇高的敬意和衷心的感谢！

　　在肯定成绩的同时也清醒地看到，我们的工作与自由贸易试验区和中国特色自由贸易港的建设要求、与人民群众日益增长的美好生活需要仍有差距。对外开放、改革创新的力度、深度和广度还不够，营商环境有待进一步改善；实体经济不强，基础设施建设滞后，人才资源相对匮乏，发展质量和效益有待进一步提升；居民收入不高，物价尤其是菜价问题依然突出，交通、教育、医疗等公共服务短板有待进一步补齐；激励干部担当的措施不多，一些公职人员“隐性不作为”的情况屡有发生，体制机制活力有待进一步释放。对这些问题，我们将采取有力措施加以解决。

　　2019年工作安排

　　2019年是新中国成立70周年，是决胜全面建成小康社会的关键之年，是省委省政府确定的全面深化改革开放政策落实年，做好今年的工作，十分重要。根据市委部署，今年政府工作的总体要求是：以习近平新时代中国特色社会主义思想为指导，全面贯彻落实党的十九大和十九届二中、三中全会精神，统筹推进“五位一体”总体布局，协调推进“四个全面”战略布局，坚持稳中求进工作总基调，坚持新发展理念，坚持推进高质量发展，坚持供给侧结构性改革，坚持深化市场化改革、扩大高水平开放，以贯彻习近平总书记“4·13”重要讲话和中央12号文件精神为主线，以建设自由贸易试验区和中国特色自由贸易港为统领，紧紧围绕“三区一中心”战略定位，统筹推进稳增长、促改革、调结构、惠民生、防风险工作，提高人民群众获得感、幸福感、安全感，加快建设国际化滨江滨海花园城市，着力打造海南争创中国特色社会主义生动范例的引领区和示范区。

　　主要预期目标是：全市生产总值增长7.5%—8%，固定资产投资增长10%左右，社会消费品零售总额增长10%左右，地方一般公共预算收入增长9%左右，城乡常住居民人均可支配收入增长8.5%左右，居民消费价格涨幅控制在3.8%以内，城镇登记失业率控制在3%以内，全面完成省下达的节能减排降碳和安全生产控制目标。

　　围绕上述目标，我们将着力抓好以下6个方面、26项工作。

　　（一）站在更高起点谋划和推进改革开放，打造全面深化改革开放试验区的创新区

　　高举改革开放旗帜，实施更加积极主动开放战略，持续推进制度创新，大力实施“六大工程”，让海口成为新时代全面深化改革开放试验区的排头兵。

　　实施江东新区先导工程。落实“两年出形象、三年出功能、七年基本成形”要求，年度计划投资300亿元以上。坚持综合考虑、长远谋划，体现生态特色，全面完成江东新区控制性详细规划及地下空间、交通、市政管网、生态等专项规划。加快起步区建设，推进哈罗公学、音乐厅、美术馆等公共配套服务集中布局，启动地下空间综合开发；年内启动10家以上总部大楼建设，积极引进国内外企业总部和功能性机构落户，打造总部经济聚集区。加速推进临空产业园开发，全面推进整体拆迁安置和“五通一平”，完善园区管理体制，争取增设海关特殊监管区域，启动“一站式”飞机维修服务基地建设，大力培育综合物流、保税维修等国际化自贸产业体系，形成海口新的经济增长极。加快文明东越江隧道、白驹大道延长线等骨干路网和供水、污水处理等基础设施建设，率先推广人脸识别、无人驾驶等新技术运用，构建生态智慧交通体系。继续加强规划、土地、户籍等管控，确保新增违建为“零”。重点引导特色小镇和美丽乡村建设，探索开展“三块地”改革，创新社区治理模式，打造城乡融合发展示范。加强江东历史文脉保护和传承，开展水系活化、海岸带保护、植绿复绿等生态修复工程，向世人展现“蓝绿交织、江海相映、林城相依、水城共融”的新形象。

　　实施重要片区提升工程。加强产业项目准入管控，建立科学评估论证机制。美安科技新城坚持产城融合方向，以深化与上海临港合作为突破口，重点在生物制药、低碳制造等领域引进一批高新技术企业，打造海口实体经济主战场。综保区重点开展投资贸易自由化便利化改革，探索实施保税、非保税、口岸货物同仓存储、分类监管，加快推进跨境电子商务综合试验区和国际文化艺术品保税交易中心建设，争取设立橡胶期货保税交割库，全力打造海口对外开放新高地。新海生态临港新城加快客运综合枢纽和免税城建设，争取设立海南国际航运交易所，着力打造琼州海峡经济带“桥头堡”和海南海上“新门户”。西海岸南片区全面启动路网建设、生态治理和景观提升，在中央核心带再造一个“万绿园”，围绕未来产业园和复兴城西海岸互联网总部基地两大核心，打造高科技企业第二总部、新兴社交媒体、虚拟现实与人工智能等融合发展的创新培育区。海口湾继续强化整体管控和景观提升，完善公共文化建筑和国际化高端休闲服务设施配套，建设吸引市民游客的最美城市客厅，打造成为承载市民服务功能、彰显城市活力的国际化卓越港湾。

　　实施招商引资龙头工程。坚决落实省委吸引外资十大举措，扎实开展“引进外资年”活动，全年引进和实际利用外资增长1倍以上。加强与国际咨询服务机构合作，推行外商投资企业商务备案与工商登记“一口办理”，力争全年引进世界500强、全球行业领军企业和知名品牌企业增长20%以上。围绕“三大领域”“十二个重点产业”，制定产业链招商指导目录，建立全员招商、小分队招商、“一把手”招商等工作模式，完善专业化市场化招商机制，在北京、上海、深圳等地开展驻点精准选商招商。在招商引资、签约项目落地和运营等方面严格落实项目责任制，盯牢“3·18”“5·18”“7·18”“9·18”“11·18”等五个重要节点，倒排时间计划，集中开工、签约一批重点项目，确保招商引资项目落地生效。

　　实施聚才引智工程。全面落实“百万人才进海南行动计划”，力争全年引进落户各类人才1.8万人、增长20%。构建更加开放的引才机制，推动国际人才管理改革试点，探索建立与国际接轨的全球人才招聘制度和吸引外国高技术人才的管理制度，力争引进国际一流人才100人。创新人才培养机制，全力支持海南大学等高校发展，积极引进国内外知名高校和研究机构。鼓励企业与省内外专业院校合作，联合培养重点产业急需的专业技术人才。完善正向激励机制，充分调动现有本地人才的积极性；加快实施人才安居工程，年内新增人才房6000套。完善人才“绿卡”机制，为人才提供落户、子女入学、就医社保等“一站式”服务。

　　实施营商环境优化工程。出台落实省政府优化营商环境四十条的具体实施细则，集中开展营商环境评估和优化提升行动，加快培育形成法治化、国际化、便利化的营商环境和公平统一高效的市场环境，力争年内新增市场主体6万家、注册资金4000亿元。深化“放管服”改革，积极稳妥推进政府机构改革，加快推行“证照分离”和“多证合一”改革，全面推行建设项目“极简审批”和联合验收，优化“互联网+政务服务”。推行国际贸易“单一窗口”再升级，口岸通关效率达到全国先进水平。加快投资者司法咨询服务平台建设，争取设立知识产权法庭、国际商事调解中心、涉外民商事法庭，保护所有投资者权益。积极创建国家社会信用体系建设示范城市，加快建设以信用监管为核心、与负面清单管理方式相适应的监管体系。全力支持民营企业发展，重点围绕融资难、要素保障难、政策落地难、转型升级难、办事难等瓶颈问题，集中开展纾难解困活动，让民营企业像享受新鲜空气一样享受一流营商环境。

　　实施基础设施改善工程。大力提升基础设施网络化智能化水平。加快“东西双港驱动”，美兰机场（二期）力争国庆前正式投入使用，推进美兰机场（三期）、马村港集装箱码头等项目前期工作；推动海口港与洋浦港组合发展，增加境内外海上航线，积极参与国际陆海贸易新通道建设，不断提高全球航运资源配置能力。全力推进G15沈海高速（海口段）、绕城高速（二期）和海秀快速路（二期）建设，完成轨道交通前期工作，确保海口城际公交化铁路投入运营。开展龙昆南路、灵桂大道、琼山大道等骨干路网拓展工程，打通4条以上断头路。实施5G规模组网试点，实现城乡光纤网络和高速移动通信网络全覆盖。率先开展智能电网、微电网等示范项目建设，力争全年全市户均停电时间低于10小时、中心城区低于1小时，达到省会城市中上游水平。完成南渡江引水工程建设，加快永庄水厂（三期）、江东水厂扩建及并网。统筹推进雨污分流改造工程建设，加快城市积水点改造，力争两年内解决城市内涝问题。

　　（二）加快培育发展现代化经济产业体系，打造国际旅游消费中心的体验区

　　坚决贯彻新发展理念，大力深化供给侧结构性改革，编制完善产业发展规划，加快培育发展以旅游业、现代服务业、高新技术产业“三大领域”为支撑的开放型生态型服务型产业体系，推动经济高质量发展。

　　推动旅游消费提质升级。全年接待游客人数和旅游总收入增长10%以上。拓展旅游消费空间，落实更加开放便利的入境免签、离岛免税购物、邮轮游艇等政策，开辟泛南海邮轮航线，实施琼港澳游艇自由行。抢抓海南体育旅游示范区建设机遇，出台体育产业发展政策，积极承接国际国内顶级赛事，做好帆船帆板等海上运动休闲旅游项目开发。加大文创产业主体培育，大力发展影视创作、动漫游戏、数字创意、演艺等特色产业。完善影视产业扶持政策，培育和引进一批影视企业和工作室，积极引进外资演出经纪机构1至2家。提升旅游消费质量，大力引进国内外高端酒店和管理品牌，推进海口湾国际酒吧街、骑楼美食街等特色街区建设。完善“互联网+”消费生态体系，建设“智能店铺”“智慧商圈”。加快建立与国际通行规则相衔接的旅游管理体制，提升旅游设施和旅游要素国际化水平。力争引进一批旅游龙头企业，规划建设一流的国际旅行卫生保健中心，为出入境人员提供高质量服务，力争实现境外游客增长15%以上。

　　大力发展现代服务业。制定实施服务业重点领域高质量发展行动计划，建立符合国际惯例的财政扶持培育体系。加快金融业开放创新，引进国内外银行、证券、保险等金融机构，依托自由贸易账户体系创新业务，提高企业跨境存贷汇兑便利化水平。推动会展产业发展，加快海南国际会展中心（二期）场馆建设，大力引进和培育国际知名品牌会展，办好国际新能源车展、国际商品博览会等大型国际展览会。加快商贸服务型国家物流枢纽承载城市建设，为国际国内和区域性商贸活动提供商品仓储、干支联运、分拨配送以及金融、结算、供应链管理等服务，全年货物吞吐量增长7.3%以上。加快国家电子商务示范城市和共同配送试点城市建设，推动电子商务与快递物流协调发展，积极争取设立国际热带农产品交易中心、定价中心和价格指数发布中心。继续引进国内外优质医疗资源和交流合作办医，推动医疗服务业和康养服务业快速发展。大力引进国际化的规划、建筑、设计、仲裁、知识产权等专业服务机构，全方位提升服务保障能力，争取中国（海南）知识产权交易所落户。

　　加快发展高新技术产业。抢抓新时代产业革命和技术变革先机，深入实施创新驱动发展战略，着力发展互联网、生物医药和高端装备制造等高科技产业，延伸实体经济产业链条、打造高新技术产业集群。加快“互联网+”创新领军企业培育和引进，力争全年互联网相关营业收入增长17%以上。积极推动重大新药创制国家科技重大专项成果转移转化试点落地，加快生物医药和转化医学基地建设，积极申报医药上市许可持有人制度试点城市，启动长安制药、威高特医食品等9个项目建设，力争全市医药工业总产值增长10%以上。支持金盘科技、威特电气、海马汽车等龙头企业提升竞争力。围绕物联网、虚拟现实、人工智能等新兴产业，策划一批重大信息化应用示范工程，推动与实体经济深度融合。规划建设海甸岛物联网应用创新基地。整合利用现有园区和孵化器创业空间，打造区域科技创新中心，建设海南离岸创新创业示范区。全市研究与试验发展经费投入强度达到1.5%以上，新增高新技术企业30家以上。

　　（三）加快推进国际化滨江滨海花园城市建设，打造国家生态文明试验区的展示区

　　牢固树立社会主义生态文明观，实行最严格的生态环境保护制度，全面开展生态文明建设、乡村振兴、城市更新“三大行动”，为全国生态文明建设作出表率。

　　扎实开展生态文明建设行动。深入推动中央环保督察、国家海洋督察反馈意见整改，贯彻落实省委三十条生态环保措施。建立“多规合一”基础上的自然资源统一监管体系，严格执行党政领导干部自然资源资产离任审计、生态环境损害责任追究制度。健全环保信用评价等体制机制，建立环境污染“黑名单”制度。开展第三次国土调查和自然资源资产产权登记试点工作。坚决打好污染防治攻坚战。启动建设领先全国、覆盖全市的大气、水、土壤“三位一体”的环境监测监控系统。扎实推进生态环境六大专项整治，持续保持打违控违高压态势，确保完成年度“两违”整治任务。继续加大打击非法采砂力度，多措并举保障砂石供应。强化大气防治治理，确保空气质量优良率始终保持在98%以上。推进陆海统筹，持之以恒抓好龙昆沟等32个水体的常态化治理，近岸海域水质保持优良。深入开展城乡环境综合整治，推进生活垃圾分类，确保通过国家卫生城市年度复核验收。狠抓农业面源污染防治，大力实施化肥农药“减施工程”。开展以红树林保护为重点的湿地保护修复行动，争取五源河、美舍河国家湿地公园通过国家验收，加快三江红树林、潭丰洋等省级湿地公园建设。践行绿色生产生活方式。加快构建绿色产业体系，建立绿色产品政府采购制度。禁止一次性不可降解塑料袋、塑料餐具的生产、销售和使用，加快推动快递业绿色包装应用与回收。鼓励推广装配式建筑。大力倡导和推进绿色出行，实施公交优先战略，公交出行分担率提高3个百分点以上。

　　扎实开展乡村振兴战略行动。按照“产业兴旺、生态宜居、乡风文明、治理有效、生活富裕”总体要求，深化农业供给侧结构性改革，大力发展设施农业、特色农业和品牌农业。建立标准化示范基地1.2万亩，加快火山荔枝、火山石斛、大坡胡椒等地理标志证明商标申报。大力推进农产品加工和现代物流园区规划建设，深化供销合作社综合改革，搭建农业社会化服务体系。深入推进百镇千村工程，抓好农村人居环境整治，加快农村公路“六大工程”建设，实施农村生活污水治理全覆盖工程，推进农村供水管网、燃气管网建设。加强镇村风貌管控，全面完成村庄规划编制，推进农房逢建必报。注重传统村落保护和文化传承，打造一批体现热带风情的精品小镇，建设美丽乡村33个。加快田园综合体示范项目建设，支持斌腾石斛等创建共享农庄试点。开展全国农村社区治理试验区创建。培养造就一支懂农业、爱农村、爱农民的“三农”工作队伍。深化农村集体产权制度改革，年内全面完成全市镇、村、组三级农村集体资产的清产核资和所有村集体经济组织成员身份确认工作，支持各区试点开展“三块地”改革。发展新型农业经营主体，促进农户与现代农业发展有机衔接，让农民合理分享全产业链增值收益。

　　扎实开展城市更新行动。做好城市总体规划修编。加强沿海沿江沿河沿湖沿路等“五沿”重点区域的建筑风貌管控，全力抓好龙昆路等6个综合提升项目建设。进一步强化广告牌管理，规范城市道路命名，提升标识导识的国际化水平。加快推进五源河文体中心（二期）、科技馆、博物馆、图书馆等公共基础设施建设。继续大力推广种植大叶油草、三角梅、椰子树等本地花草树木，鼓励新城区和新建筑立体绿化。加快推进金牛岭公园改造、海秀公园建设，新建5个城市小游园。深度做好骑楼老街和府城片区的修缮保护、文化发掘和业态调整，全面推进五公祠、丘濬、海瑞等历史文化公园建设提升以及“石室仙踪”石刻保护和遗存发掘，加快明昌塔、文峰塔文化公园建设。还海还岸还景于民，举全市之力实施海口湾畅通工程，让老百姓共享最好的空间资源，打造宜居宜业城市的全省示范。

　　（四）持续加强支撑保障能力建设，打造国家重大战略服务保障区的示范区

　　更好服务海洋强国战略。加快国家海洋经济创新发展示范城市建设，举办中国（海南）海洋产业博览会，强化海洋研究机构与重点实验室建设，大力发展海洋交通运输、海洋制药和海洋高端装备制造；积极引进南海资源开发企业设立区域总部和营运中心，开展航运保险、海损理算、船舶融资租赁等高端航运服务。加快推动现代化海洋牧场示范基地建设。

　　更好服务“一带一路”倡议。用好用足与“一带一路”沿线国家和地区包括第五航权在内的航权政策，吸引国内外航空公司开辟洲际航班，加密与香港、新加坡等地商务航班。加快与东盟沿海国家海上集装箱航线覆盖，开辟跨国多站式邮轮航线，推动泛南海旅游经济合作圈建设。加快建设21世纪海上丝绸之路重要战略支点，深化与“一带一路”沿线国家和地区在设立境外总部、旅游、海洋渔业、人文交流等领域的交流合作。加强与海外侨社联系，鼓励海外侨胞参与“一带一路”建设。

　　更好服务区域协调发展战略。积极融入粤港澳大湾区，加强泛珠三角区域合作，推动北部湾城市群建设，促进港航、旅游协同发展。着力提升琼州海峡客滚运输服务水平，推进湛江至海口高铁（含轮渡）等项目前期工作，推进湛江、北海、桂林等地水上飞机线路开通，打造琼州海峡“半小时立体交通圈”。更好发挥省会中心城市的带头、引领、示范作用，立足全岛同城化目标，加快“海澄文”一体化进程，优先推动基础设施一体化，谋划轨道交通、城际铁路等项目规划建设；在产业发展差异化、生态环境协同化、公共服务同城化等方面实现突破。

　　（五）着力提高保障和改善民生水平，打造人民群众美好生活的幸福之城

　　牢固树立以人民为中心的发展思想，着力抓好“五大任务”，为海口全面建成小康社会收官打下决定性基础。

　　坚决打赢精准脱贫攻坚战。年内实现存量贫困人口345户849人全部脱贫，已脱贫人口稳固脱贫不返贫，22个已脱贫贫困村实现稳固，在全市消除绝对贫困。继续从源头抓好贫困户识别、帮扶和退出工作，努力在提高精准度、降低返贫率上走在全省前列。突出抓好产业帮扶和转移就业，继续推进脱贫攻坚项目库和消费扶贫活动，确保组织化产业帮扶全覆盖、贫困家庭至少1名劳动力稳定就业。加快完善综合保障体系，集中力量解决“三保障”、安全饮水等重点难点问题。坚持扶贫和扶智相结合，扎实开展道德扶贫、法制扶贫、文化扶贫，在全社会形成诚实守信、勤劳致富的正能量。

　　优先发展教育事业。持续推进“一校两园”建设，年内新建公办幼儿园10所、公办中小学校10所，新增学位2.52万个。全面实施基础教育提质工程，继续加大优质教育资源的引进，大力推进中小学教师“区管校聘”改革，试行学区化管理，探索集团化办学，推行委托管理、强校带弱校、学校联盟等办学形式，让家门口的好学校、好老师多起来。大力支持和发展好职业教育、特殊教育。充分发挥收入分配机制的激励和导向作用，在海南侨中、滨海九小等探索开展教师薪酬制度改革。年内完成公办高中学校和中考考点空调安装，力争三年内覆盖全市公办中小学。

　　大力提升医疗健康服务能力。继续全面深化公立医院综合改革，稳步推进区域紧密型医联体建设，强化提升家庭医生签约服务。深化与知名医学院校合作，打造2至3个精品学科，建成市人民医院国际医疗部。启动市第二人民医院和江东新区三级公立医院建设，规划建设市孕产妇危重症救治中心、新生儿危重症救治中心和人工辅助生殖中心。加快中医药事业发展，推动市中医院国际中医中心建设。全面完成基层医疗卫生机构标准化建设，在全省率先构建“15分钟城市健康服务圈、30分钟乡村健康服务圈”。支持社会力量提供多层次多元化医疗健康服务，扩大有效供给。

　　织密扎牢民生保障网。着力把菜价稳定作为今年提高群众满意度的重要民生工作来抓，全面落实“菜十条”，多措并举保供稳价。落实就业优先政策，促进高校毕业生、农民工等群体就业创业，全年城镇新增就业3.3万人以上。全面实施全民参保计划，稳步提高城乡居民基础养老金标准。鼓励发展商业健康保险，完善多层次医疗保障体系。落实城乡低保制度，精准推进“两项制度”衔接，逐步提高社会救助标准，切实保障困难群众基本生活。加强养老服务设施规划建设，推进“长者饭堂”社区全覆盖。推进残疾人无障碍环境建设。做好退役军人安置、管理和保障工作。积极推进利用集体建设用地建租赁住房试点，加快完善面向本市居民和引进人才的住房保障和供应体系。加快江东新区等新建区域直饮水建设。全面保障粮食安全。坚持问需于民、问效于民，全力以赴办好省、市民生实事。

　　加强和创新社会治理。深入开展社会文明大行动，持续巩固提升全国文明城市创建成果，推进新时代文明实践中心向基层延伸和覆盖，打造百姓家门口的服务中心、慈善中心、信仰中心。实施文化惠民工程，落实全民健身战略，全面完善公共文化和体育服务设施。加快“智慧海口”建设，推动市民游客中心“城市形象展示中心、便民利民服务中心和城市综合管理中心”三大功能提升，巩固和强化“12345+网格化”和“椰城市民云”功能。推进全民普法，构建公共法律服务实体平台、热线平台、网络平台互联互通融合发展体系。推进社会组织孵化基地建设，建立政府购买服务机制。加强物业服务行业诚信管理。深入实施安全发展战略，启动国家安全发展示范城市创建。大力开展停车场管理行业整治，集中研究解决学校（幼儿园）周边停车不安全、不便捷问题。加强市场综合监管，创建国家食品安全示范城市。深入推动绿色殡葬改革，同步启动市、区公共墓地规划建设。全面推进海陆空立体化治安防控新体系建设，深化扫黑除恶专项斗争，严厉打击“菜霸”“砂霸”“运霸”，全面打赢禁毒三年大会战。

　　继续做好统计、人防、地震、气象、三防、档案、史志、科协、社科联、工商联、保密、老龄、慈善、对台、海防口岸、民族宗教等各项工作，充分发挥工会、共青团、妇联、残联等人民团体的桥梁纽带作用。

　　（六）不断提升现代化治理体系和治理能力，打造人民满意政府

　　全市政府系统坚持以党建为引领，“干”字当头、“实”字为要，咬定目标不放松，强化执行抓落实，凝心聚力打造人民满意政府。

　　着力打造学习型政府。让学习成为政府从业人员的一种生活方式，建立健全政府班子集体学习制度，定期举办领导干部集中学习专题讲座，鼓励在职干部参加继续教育，加快建设高素质专业化干部队伍。在规划、司法、金融、审计等专业性较强的政府机构设置高端特聘职位，实施聘期管理和协议工资。加强与国内发达地区的公务员学习交流，开展公务员国际交流合作，稳妥有序开展公务人员境外培训。

　　着力打造实干型政府。坚持说实话、谋实事、出实招、求实效，狠抓政府执行力建设。实行重点任务清单管理，明确责任，倒排工期，挂图作战，全程督办，让“马上就办”“钉钉子”“真抓实干”成为各级干部的行动自觉。强化斗争精神，积极防范化解政府债务、金融、房地产等各类风险。优化政府绩效考核，深化国资国企、财税金融体制改革，建立完善激励机制和容错纠错机制，营造干事创业的良好氛围。

　　着力打造法治型政府。健全依法决策机制，全面落实政府法律顾问制度，积极推进各级政府及其职能部门法律顾问全覆盖，每半年开展一次政府决策第三方评估。全面推行行政执法公示制度、执法全过程记录制度、重大执法决定法制审核制度。强化政策性文件的合法性与合理性审查，提高政策的连续性和可操作性。主动接受人大的法律监督、工作监督和政协的民主监督，广泛接受社会监督和舆论监督。

　　着力打造诚信型政府。坚持以诚信为本，言必行、行必果。加快政府守信践诺机制建设，探索建立政务和行政承诺考核制度，坚决防范和解决招商引资协议不落实、优惠政策不兑现、法院判决和行政复议不执行等问题。积极构建政府与法院、检察院等单位的失信惩戒联动机制和信息共享平台，加大对侵犯知识产权、制假售假、商业欺诈、逃债骗贷等不诚信行为的打击力度。大力推进政务公开，努力建设“最阳光政府”。

　　着力打造廉洁型政府。坚持把全面从严治党贯穿政府自身建设的全过程，持之以恒落实中央八项规定精神，深度抓好省委巡视反馈问题整改，驰而不息纠正“四风”。严格落实党风廉政建设责任，加强廉政风险防控，持续加大公共资金、国有资产、国有资源监管，规范公共资源交易和招投标管理，扎牢制度的笼子。加强重点项目和资金审计监督，坚决打击发生在群众身边的腐败和不正之风。

　　各位代表，让我们更加紧密地团结在以习近平同志为核心的党中央周围，在省委省政府和市委的坚强领导下，发扬敢闯敢试、敢为人先、埋头苦干的特区精神，励精图治、奋发有为，以优异成绩向中华人民共和国成立70周年献礼！
